# Prospective observational study to evaluate the clinical and biological safety profile of pyronaridine–artesunate in a rural health district in Burkina Faso

**DOI:** 10.1002/prp2.987

**Published:** 2022-07-19

**Authors:** Toussaint Rouamba, Paul Sondo, Isidore W. Yerbanga, Adelaide Compaore, Maminata Traore‐Coulibaly, Franck S. Hien, Nassirou A. Diande, Innocent Valea, Marc Christian Tahita, Rita Baiden, Fred Binka, Halidou Tinto

**Affiliations:** ^1^ Clinical Research Unit of Nanoro, Institute for Research in Health Sciences National Center for Scientific and Technological Research Ouagadougou Burkina Faso; ^2^ INDEPTH–Network Accra Ghana

**Keywords:** artesunate, Burkina Faso, cohort event monitoring, hepatic safety, pyronaridine, safety monitoring

## Abstract

The assessment in real‐life conditions of the safety and efficacy of new antimalarial drugs is of greatest interest. This study aimed to monitor and evaluate both clinical and biological safety of pyronaridine‐artesunate (PA) in real‐life conditions in Burkina Faso's health system. This was a single‐arm, open‐label study, where patients attending Nanoro health facilities with uncomplicated malaria were consented to be part of a cohort event monitoring (CEM). At inclusion (day‐0), PA was administered orally once a day for 3 days. Patients spontaneous reported any clinical adverse events (AEs) occurring within 28 days following the treatment. Additionally, the study focused on AEs of special interest (AESI), namely clinical signs related to hepatotoxicity and increased alanine aminotransferase (ALT) and aspartate aminotransferase (AST). A nested subset of patients with blood sample collection at day‐0 and day‐7 were monitored to investigate the effect of PA on biochemistry parameters. From September 2017 to October 2018, 2786 patients were treated with PA. About 97.8% (2720/2786) of patients did not report any AE. The most commonly reported events were respiratory, thoracic, and mediastinal disorders (8.3 per 1000), infections and infestations (7.9 per 1000), and gastrointestinal disorders (7.2 per 1000). No clinical or biological hepatotoxicity event related to PA was reported during the follow‐up. Changes in biochemistry parameters remained within laboratory reference ranges. The study showed that PA is a well‐tolerated drug and should be considered as a good option by malaria control programs in countries where existing first‐line antimalarial drugs are continuously threatened by the emergence of drug resistance.

**Abbreviations**
ACT: artemisinin‐based combination therapies
ADRs: adverse drug reactions
AEs: adverse events
AESI: AEs of special interest
AL: artemether‐lumefantrine
ALT: alanine aminotransferase
ASAQ: artesunate‐amodiaquine
AST: aspartate aminotransferase
CEM: cohort event monitoring
DHA‐PQP: dihydroartemisinin‐piperaquine
HCWs: health care workers
HDSS: Health and Demographic Surveillance System
MMV: Medicines for Malaria Venture
NMCP: National Malaria Control Program
PA: pyronaridine–artesunate
SMC: Seasonal Malaria Chemoprevention
SSA: sub‐Saharan Africa

## 1 BACKGROUND

With the contribution of international organizations such as Medicines for Malaria Venture (MMV) and other public–private partnerships, new effective and well‐tolerated antimalarial drugs for large‐scale use are getting available on the market.^1^ These new antimalarial drugs, as well as those already on the market, were developed to provide countries with rational approaches for decision‐making on the prioritization, selection, and adoption of antimalarial treatment that is appropriate for their health systems.^2^ In addition to planning the deployment of these new interventions, it is also important to consolidate their safety of use under real‐life conditions.^3^ Traditionally, Phase I, II, and III controlled and randomized clinical trials conducted by researchers are usually well funded and provide baseline data on antimalarial drugs efficacy and safety before they are marketed. In sub‐Saharan Africa (SSA), these new antimalarial drugs are often introduced into health systems through national policy decisions, often based on data from Phase I, II, and III trials. Nevertheless, the identification of rare or non‐detected adverse drug reactions (ADRs) during the drug development process and the establishment of the efficacy and safety of these drugs are achieved through post‐marketing surveillance. However, due to the poor performance of well‐established pharmacovigilance systems in malaria‐endemic countries, it is very difficult for SSA countries to set up large‐scale Phase IV studies for drug monitoring in real‐life conditions to guide national policy decisions.^4^, ^5^, ^6^, ^7^

In Burkina Faso, since 2008, fixed‐dose artemisinin‐based combination therapies (ACTs) were adopted as treatment for uncomplicated malaria.^8^ Based on data available (cost‐effectiveness) on these ACTs, artesunate‐amodiaquine (ASAQ), artemether‐lumefantrine (AL), and dihydroartemisinin‐piperaquine (DHA‐PQP) were adopted by policies makers as first‐line drugs for the treatment of uncomplicated malaria.^8^ Currently, with the scaling‐up of Seasonal Malaria Chemoprevention (SMC) among children under 5 years of age in the country, the treatment of uncomplicated malaria in this age group should no longer include either amodiaquine or combination drugs containing amodiaquine, especially during the high transmission period of malaria (SMC period).^9^ In such context, AL and DHA‐PQP remain the available drugs for the management of uncomplicated malaria cases. A recently published study seemed to point out an inadequate efficacy of AL at day 28.^10^ This result should be taken with caution as another publication, concluded that there is no convincing evidence in the articles reviewed that multidrug resistance has emerged in Burkina Faso, in particular amodiaquine, lumefantrine, and piperaquine resistance.^11^ In addition to conducting urgently other studies (with the recommended monitoring and quality control of slide reading^11^) to confirm or refute the findings on the emergence of plasmodium resistance to AL, it is also crucial and urgent to find an alternative drug to reduce the therapeutic pressure on AL and DHA‐PQP (an important source of antimalarial drug resistance) and to replace ASAQ in order to allow the diversification of first‐line malaria treatments. Pyronaridine–artesunate (PA) a fixed‐dose ACT, represents the first ACT to have been validated by a stringent regulatory authority for the treatment of *Plasmodium falciparum* and blood‐stage *P. vivax* according to multicenter clinical studies in Africa and Asia.^12^ Randomized controlled trials carried out in malaria‐endemic countries to evaluate the efficacy of ACTs in the treatment of uncomplicated *P. falciparum* demonstrated that PA is equivalent to the other marketed ACTs.^13^, ^14^ Furthermore, these studies showed that PA is generally well tolerated.^13^, ^14^, ^15^, ^16^, ^17^ The safety warning that was reported with the PA use was a transient increase of hepatic transaminases,^13^, ^14^, ^16^, ^17^ without any clinical signs or symptoms of hepatotoxicity.^13^, ^18^, ^19^ Therefore, WHO recommends malaria‐endemic countries to include PA in their national treatment guidelines.^12^ However, this deployment should be conducted under a strong pharmacovigilance system as required for the introduction of all new medicines.^3^ Such data are required to guide National Malaria Control Program (NMCP) for the adoption of new ACTs and to provide more option for prescription, especially in areas where there is more than one ACT in the policy. To fill the gap of this shortage of safety data, this study aimed at monitoring and evaluating both the clinical and biological safety of PA used in real‐life conditions in the health system in Burkina Faso. In other words, there were a primary objective that consisted to evaluate the clinical safety of PA when used under usual conditions among patients with uncomplicated malaria, and a main secondary objective that consisted of an intensive assessment of a nested subset of patients (nested cohort or active group) to evaluate the effect of the administration of PA on blood biochemistry parameters.

## 2 PATIENTS AND METHODS

### 2.1 Study setting

The study was carried out from September 2017 to October 2018 in the health district of Nanoro through the Health and Demographic Surveillance System (HDSS).^20^ The HDSS of Nanoro is on the INESS (INDEPTH Effectiveness and Safety Studies) platform and has a good experience in effectiveness and safety studies on antimalarial drugs.^21^, ^22^ The HDSS area is located in rural setting, Central‐west of the country. The Nanoro HDSS covers a catchment area of 594.3 km^2^ and lies between longitudes 1°892 537 and 2°83 146 West and latitudes 12°857 955 and 12°872 863 North. Malaria is hyper‐endemic and peaks during the rainy season (July to November). The number of inhabitants covered by the HDSS was estimated at about 60 000 in 2018, of whom 20% were children under 5 years of age. At the same period, malaria represented the top one of all diseases, accounting for about 41.3% of outpatient medical consultations and 57.0% of hospitalizations. Children under 5 years of age are the most vulnerable group affected by malaria, accounting for 58.5% of all malaria cases. In addition, the study area is characterized by a marked seasonality of malaria transmission^23^ making it an appropriate place for SMC implementation. Currently, ASAQ, AL, and DHA‐PQP are the first‐line treatments for uncomplicated malaria and ASAQ and AL are provided free of charge to children less than 5 years of age.^24^ PA is the latest ACT to be registered in the Burkina Faso health system. From a previous study, the complete adherence level to antimalarial drugs in the Nanoro HDSS area is satisfactory and was estimated at 86%.^25^

### 2.2 Study design and patients

This was a single‐arm, open‐label observational, non‐comparative phase IV study. All patients regardless of gender and age attending the health facilities in the Nanoro HDSS catchment area and for whom a diagnosis of uncomplicated malaria was posed (or suspected) by the public sector health care workers (HCWs) were invited to participate in the study. Patients were consented to be part of a cohort event monitoring (CEM). All consented participants (main cohort) were asked to return voluntarily to health facilities and to spontaneously report any occurrence of adverse events. Eligible patients were aged over 6 months, weighed more than 5 kg and were able to take oral medications, and signed informed consent (a parent or guardian consented for children below 18 years old). Patients were excluded if they had any of the following: the presence of clinical signs or symptoms of hepatic injury (such as nausea and abdominal pain associated with jaundice) or known to have severe liver disease (i.e., decompensated cirrhosis, Child‐Pugh stage 3 or 4); known to be pregnant or lactating; severe malaria; known allergy to artemisinin and/or to pyronaridine. A nested subset of patients (nested cohort) with blood sample collection at day‐0 and day‐7 were monitored to investigate the effect of PA on blood biochemistry parameters.

Shin Poong Pharmaceuticals Limited donated PA (Pyramax for brand name) but did not have any role in reviewing the protocol or the manuscript.

### 2.3 Enrolment and study procedures

At the enrolment visit (day 0), in both groups, data on medical and drug history were obtained from each eligible patient and were recorded on an individual case report form (CRF). For each patient, a detailed clinical examination was performed by the HCWs and the findings were recorded on the appropriate CRF. Data on concomitant medications for each patient were also collected.

Patients included in both passive and active monitoring cohorts were requested to return voluntarily to health facilities and to spontaneously report any AEs occurring within 28 days after the administration of the first dose of PA, whereas patients included only in the active monitoring cohort had a scheduled follow up visit at day 7 (±2 days) at health facility and home visit at day 28. For the latter group, data on clinical conditions and concomitant treatments were collected at each scheduled visit. Additionally, blood smears for thick and thin film and blood spot was systematically obtained on days 0 before drug administration and day 7 after drug administration. Venous blood samples were collected on day 0 before drug administration and day 7 after drug administration. Then, plasma samples were processed to investigate specific liver function tests (LFTs), namely Alanine aminotransferase (ALT), Aspartate aminotransferase (AST), total bilirubin, as well as renal function parameters namely creatinine and urea. Blood glucose was also measured.

In case of unscheduled visit or whether the patient returned voluntarily to spontaneously report an adverse event within the 28 days after PA administration, data on clinical condition and concomitant treatments were collected. Additionally, blood smears for thick and thin film were collected and a venous blood sample was drawn for biological investigation according to the clinical judgment of the study physician.

Although in Burkina Faso ACTs are recommended for pregnant women in the second and third trimester of pregnancy,^8^ female patients were encouraged to inform the study team if they get pregnant within a period of 2 months after the start of the PA treatment.

### 2.4 Study drug administration

For enrolled patients, PA was administered every 24 h from the first administration for 3 days. The first dose (day 0) was administrated under direct supervision of a HCW at the health facilities. After drug intake, patients were observed 60 min. For those who vomited within 30 min, a complete dose was re‐administered, whereas for those who experienced vomiting within 30–60 min, a half dose was re‐administered. Re‐administration was attempted only once. Then, HCW explained to the patients how to take the second and third doses at home after 24 h (day 1) and after 48 h (day 2) from the initiation of the treatment. PA was administered according to the patient body weight. Two types of presentation of PA were used to facilitate the dosing and administration: sachet (granules) for children under 20 kg and tablets for children and adults weighting more than 20 kg. The tablet presentation was dosed at 180/60 mg of PA, whereas the sachet presentation was dosed at 60/20 mg of PA. Daily dosing according to the weight is shown in Table 1.

**TABLE 1:** Daily doses of PA administered according to patient weight

Body weight (kg)	Daily dose (mg)	Number of sachets per dose	Number of tablets per dose
5 to <8	20/60 PA	1	
8 to <15	40/120 PA	2	
15 to <20	60/180 PA	3	
20 to <24	60/180 PA		1
24 to <45	120/380 PA		2
45 to <65	180/540 PA		3
≥65	240/720 PA		4

### 2.5 Follow‐up for detection of adverse events

Patients in the active monitoring cohort had a scheduled follow‐up visit on day 7 (±2 days) at the health facility. During this visit, the treatment adherence, that is, patients who complied with the recommended treatment according to age, was verified retrospectively through self‐reporting. Additionally, the occurrence of an AE between the drug intake and the day of the visit was reported by the HCWs. A check‐in was performed to detect whether symptoms of malaria persisted or whether an AE had occurred since the first administration of PA. Patients were contacted again on day 28 to ascertain the AE recovery or to notify any further new AE. Apart from these scheduled follow‐up visits in the active cohort, similarly to those included in the passive group, patients were encouraged to return to the health facility to report any occurrence of AE. AEs were documented by the clinical team as described by the participant or caregiver. Information was reviewed and coded using the Medical Dictionary for Regulatory Activities (MedDRA version 22.1 September 2019) system organ class (SOC).

Adverse event was defined in accordance with the International Conference of Harmonization (ICH) guidelines for Good Clinical Practice (GCP) as: any untoward medical occurrence, irrespective of its suspected relationship to the study medications.^26^

At each contact, patients were assessed according to a standardized checklist, and information about current signs and symptoms was collected, including the start and end date. A severity grading scale (mild, moderate, severe, or life‐threatening), was used to grade severity of all symptoms in accordance with the toxicity grading scales developed by the WHO (Toxicity Grading Scale for Determining the Severity of Adverse Events) and the National Institutes of Health (NIH), Division of Microbiology and Infectious Diseases.^27^ The relationship (suspected, not suspected) of any AEs with PA was established, as well as their outcome. The notification of the occurrence of serious adverse events (SAE) regardless of their relationship with PA, was reported to the national ethics committee, the Drug Regulatory Authority (via the national pharmacovigilance office), and to the study sponsor within a period of 24 h.

### 2.6 Study outcome

The study's main outcome of interest was both clinical and biological safety within the 28 days after starting PA treatment. The clinical outcome (incidence of clinical AE) was evaluated through the analysis of the AEs captured by the study clinical team; whereas biological safety was defined as any significant change in liver transaminases values (elevated AST/ALT). Safety was assessed in all patients who received at least one dose of PA. Special attention was given to the AEs classified as severe and/or adverse events of special interest (AESI) related to hepatotoxicity (jaundice, dark urine, putty/mastic stool, worsening of fatigue, nausea, vomiting, anorexia, abdominal pain, itching, rash, spontaneous bruising, or appearance of red spots). Serious liver reactions and hypersensitivity were also considered as an outcome of interest (AESI).

Safety outcomes also included any significant change in the total bilirubin, creatinine, and urea values.

### 2.7 Data management and analysis

For the CEM for the active or nested cohort, all visit data were captured using paper CRF and then double‐entered and verified using a database designed under an open‐source software, OpenClinica. For the CEM for passive or main cohort at enrolment visit, data were collected during the medical examination using an electronic CRF developed on open‐source software, Open Data Kit (ODK) Collect and, in case of unscheduled visit, data were captured with paper CRF and then double‐entered on OpenClinica.

Statistical analyses were performed using R software (R Foundation for Statistical Computing, Vienna, Austria). Descriptive analyses for the total participants and for each cohort were performed as required. In this present analysis, the estimates of the incidence of each AE were performed based on crude rates, with no attempt to carry out ca ausality assessment of individual cases. For the biological safety assessment i.e. the blood biochemistry sample analysis, Wilcoxon matched‐pairs signed‐rank test was used to compare pre‐treatment (day 0) and post‐treatment (day 7) values in each parameter. This comparison was performed in all participants who had laboratory results at both day 0 and day 7.

## 3 RESULTS

### 3.1 Cohort composition and baseline characteristics

A total of 2832 patients with suspected uncomplicated malaria were screened to be enrolled in the study, and 2786 (98.4%) patients were included. Of these, 1761 agreed to be in the main cohort (passive group), whereas 1025 agreed to be in a nested cohort (active group). Table 2 summarizes the baseline characteristics of the study population. The majority of patients enrolled were children under 5 years of age who represented more than half of the study population, 54.1% (1508/2786). At baseline, about 69.2% (1929/2786) and 9.7% (270/2786) of patients had fever (axillary temperature ≥ 37.5°C) and episode of vomiting, respectively. Malaria infection was diagnosed by rapid diagnostic test in 90.8% of patients and 9.2% of patients were diagnosed with presumptive diagnosis of malaria. The mean values of the biochemical tests (ALT, AST, bilirubin, creatinine, urea, and glycemia) performed at inclusion were within the normal ranges. However, 11 (1.2%) and 43 (4.5%) patients had ALT and AST >2× the upper limit of normal (ULN), respectively, without signs of hepatic injury.

**TABLE 2:** Demographic and baseline clinical characteristics of participants who took at least one dose of PA and completed study procedures

Characteristic	Overall	Type of cohort	
		Passive	Active
Count	2786	1761	1025
Gender, *n* (%)			
Female	1453 (52.2)	889 (50.5)	564 (55.0)
Male	1333 (47.8)	872 (49.5)	461 (45.0)
Age in year			
Mean (SD)	9.4 (13.3)	8.7 (13.4)	10.4 (13.2)
Age group, *n* (%)			
<5	1508 (54.1)	1081 (61.4)	427 (41.7)
5 to <12	646 (23.2)	311 (17.7)	335 (32.7)
12 to <18	209 (7.5)	100 (5.7)	109 (10.6)
≥18	423 (15.2)	269 (15.3)	154 (15.0)
Weight in kg			
Mean (SD)	22.6 (18.2)	21.9 (18.9)	23.8 (16.8)
Weight group, *n* (%)			
5 to <8	180 (6.5)	136 (7.7)	44 (4.3)
8 to <15	1179 (42.3)	836 (47.5)	343 (33.5)
15 to <20	419 (15.0)	224 (12.7)	195 (19.0)
20 to <24	222 (8.0)	120 (6.8)	102 (10.0)
24 to <45	308 (11.1)	137 (7.8)	171 (16.7)
45 to <65	376 (13.5)	223 (12.7)	153 (14.9)
≥65	102 (3.7)	85 (4.8)	17 (1.7)
Axillary temperature in °C			
Mean (SD)	37.9 (1.0)	37.9 (1.0)	38.0 (1.0)
Fever (≥37.5°C), *n* (%)	1929 (69.2)	1172 (66.6)	757 (73.9)
Vomiting in the previous 24H, *n* (%)			
No	2515 (90.3)	1602 (91.0)	913 (89.1)
Yes	270 (9.7)	158 (9.0)	112 (10.9)
Malaria diagnosis, *n* (%)			
Positive to *RDT‐HRP2*	2530 (90.8)	1515 (86.0)	1015 (99.0)
Presumptive diagnosis	256 (9.2)	246 (14.0)	10 (1.0)
Positive microscopy	—	831 (81.6)	831 (81.6)
Parasite density/μL *(P. falciparum*)			
Geometric mean (SD)	—	—	14 428 (2.4)
Median (Q1–Q3)		—	30 848 (3183–88 256)
ALT (IU/L), Median (Q1–Q3)		—	14.0 (10.0–19.0)
AST (IU/L), Median (Q1–Q3)		—	33.0 (25.0–43.2)
ALT >2 × ULN, *n* (%)		—	11 (1.2)
AST >2 × ULN, *n* (%)		—	43 (4.5)
Total bilirubin (μmol/dL), Median (Q1–Q3)		—	14.9 (9.5–22.9)
Creatinine (μmol/L), Median (Q1–Q3)		—	35.8 (28.4–49.1)
Urea (μmol/L), Median (Q1–Q3)		—	3.5 (2.6–4.6)
Glycemia (mmol/L), Median (Q1–Q3)		—	4.7 (3.6–5.7)

Abbreviations: ALT, alanine aminotransferase; AST, aspartate aminotransferase; IQR, Interquartile range; Q1, First quartile; Q3, Third quartile; ULN, upper limit of normal.

### 3.2 Adverse events following the administration of the first dose (within 1 h after drug administration)

No patient was excluded at day 0 for repeated vomiting after drug intake. However, 4.3% (121/2786) of all patients experienced a vomiting episode within 1 h after drug administration at day 0. Among patients who experienced an episode of vomiting, about, 91.7% (111/121) vomited within 30 min after drug taken, whereas, 8.3% (10/121) vomited between 30 and 60 min after drug administration. Table 3 shows the repartition of vomiting episodes that occurred within an hour after study drug administration. Among all subset patients who vomited after drug intake, approximatively, 70% (81/121) had noticed a history of vomiting during the last 24 h. Apart from episodes of vomiting, no other adverse events were reported. In terms of vomiting episodes by weight bands, there was more vomiting in the young (i.e., children weighing less than 15 kg) compared to the older individuals (Table 3).

**TABLE 3:** Episodes of vomiting occurring within 1 h after drug administration

	Overall	Passive	Active
Vomiting following the intake of the first dose, *n*/*N* (%)			
No	2665/2786 (95.7)	1681/1761 (95.4)	984/1025 (96.0)
Yes	121/2786 (4.3)	80/1761 (4.6)	41/1025 (4.0)
Vomiting according to timing after drug taken, *n*/*N* (%)			
Within 30 min	111/121 (91.7)	75/80 (93.7)	36/41 (87.8)
Between 30 and 60	10/121 (8.3)	5/80 (2.3)	5/41 (12.2)
Vomiting according to weight bands, *n*/*N* (%)			
5 to <8	28/121 (23.1)	16/80 (20.0)	12/41 (29.3)
8 to <15	83/121 (68.6)	58/80 (72.5)	25/41 (61.0)
15 to <20	9/121 (7.4)	6/80 (7.5)	3/41 (7.3)
20 to <24	0/121 (0.0)	0/80 (0.0)	0/41 (0.0)
24 to <45	1/121 (0.8)	0/80 (0.0)	1/41 (2.4)
45 to <65	0/121 (0.0)	0/80 (0.0)	0/41 (0.0)
≥65	0/121 (0.0)	0/80 (0.0)	0/41 (0.0)

### 3.3 Adverse events (nausea, fatigue, abdominal pain, itching, or signs of jaundice) following pyronaridine–artesunate treatment according to the baseline ALT or AST >2 × ULN

Adverse events suggesting clinical signs and symptoms of possible hepatotoxicity, namely abdominal pain, fatigue, nausea, itching, or signs of jaundice occurred mostly among participants with normal versus abnormal baseline ALT/AST values (Table 4).

**TABLE 4:** Symptoms (nausea, fatigue, abdominal pain, itching, or signs of jaundice) following pyronaridine–artesunate treatment according to the baseline ALT or AST >2 × ULN

Variable	Overall	Baseline ALT/AST		
		Normal	Abnormal	Unknown
Nausea				
Count (%)	791	691 (87.4)	35 (4.4)	65 (8.2)
Yes	23 (2.9)	21 (3.0)	0 (0.0)	2 (3.1)
No	768 (97.1)	670 (97.0)	35 (100)	63 (96.9)
Fatigue				
Count (%)	1021	875 (85.7)	42 (4.1)	104 (10.2)
Yes	43 (4.2)	39 (4.5)	0 (0.0)	4 (3.8)
No	978 (95.8)	836 (95.5)	42 (100)	100 (96.2)
Abdominal pain				
Count (%)	792	691 (87.2)	34 (4.3)	67 (8.5)
Yes	193 (24.4)	170 (24.6)	10 (29.4)	13 (19.4)
No	599 (75.6)	521 (75.4)	24 (70.6)	54 (80.6)
Itching, or signs of jaundice				
Count (%)	1025	878 (85.7)	43 (4.2)	104 (10.1)
Yes	9 (0.9)	9 (1.0)	0 (0.0)	0 (0.0)
No	1016 (99.1)	869 (99.0)	43 (100)	104 (100)

*Note*: Normal liver function tests (LFTs) were ALT or AST ≤2 × ULN, and abnormal values of LFTs were AST or ALT >2 × ULN at baseline.

### 3.4 Reported adverse events during the 28 days of follow‐up

During the follow‐up period, the majority of patients (97.8% [2720/2786]), who received at least one dose of PA did not report any adverse event. Overall, only 2.2% (60/2786) of patients reported at least one AE in both group. About 0.4% ((7/1761) in the passive group and 5.2% (53/1025) in the active group) patients reported a total of 94 AEs (11 in the passive group and 83 in the active group). Of the reported AEs, six were classified as serious (SAEs) by the study clinicians. The frequently (cumulative incidence per 1000) reported events classified according to the MedDRA System organ classification were respiratory, thoracic, and mediastinal disorders (8.3 per 1000), infections and infestations (7.9 per 1000), gastrointestinal disorders (7.2 per 1000), general disorders (5.4 per 1000) and skin and subcutaneous tissue disorders (1.8 per 1000) (Table 5). The intensity of the majority of AEs was moderate (85.1%) and was not suspected to relate with the study drug. Only few AEs (7.4%) were graded as mild by the study physicians.

**TABLE 5:** Cumulative incidence of adverse events reported by system organ classification (grouped according to MedDRA coding) in passive (*N* = 1761), active (1025), and the total cohort (*N* = 2786)

MedDRA System organ classification	Cumulative incidence per 1000		
	Passive	Active	Overall
Respiratory, thoracic, and mediastinal disorders	3 (1.7)	20 (19.5)	23 (8.3)
Infections and infestations	3 (1.7)	19 (18.5)	22 (7.9)
Gastrointestinal disorders	2 (1.1)	18 (17.6)	20 (7.2)
General disorders	2 (1.1)	13 (12.7)	13 (4.7)
Skin and subcutaneous tissue disorders	1 (0.6)	4 (3.9)	5 (1.8)
Blood and lymphatic system disorders	0 (0.0)	4 (3.9)	4 (1.4)
Injury, poisoning, and procedural complications	0 (0.0)	2 (2.0)	2 (0.7)
Hepatobiliary disorders	0 (0.0)	1 (1.0)	1 (0.4)
Renal and urinary disorders	0 (0.0)	1 (1.0)	1 (0.4)
Others	0 (0.0)	1 (1.0)	1 (0.4)

Of the six reported SAEs, 4 (66.7%) occurred in participants under the age of 5 years. Two cases of death were recorded during the course of the study. These SAEs were not related to the PA. The details of these cases of death are provided in the Supplementary Materials.

The administrated treatment was well tolerated in general. Thirteen adverse events were assessed by study clinicians to be related to the study drug (Table 6). No serious adverse events were assessed to be related to PA. More interestingly, no hepatotoxicity event related to PA was reported during the study period. However, one patient experienced an ascites due to a splenic abscess. This adverse event was not related to PA. Another patient also presented after severe malaria, a hepatic cytolysis that investigations concluded to a cytolysis caused by malaria infection that spontaneously was resolved.

**TABLE 6:** Cumulative incidence of suspected events related to PA in passive (*N* = 1761), active (1025), and the total cohort (*N* = 2786)

MedDRA System organ classification	Cumulative incidence per 1000		
	Passive	Active	Overall
Overall number of adverse events	2 (1.1)	11 (10.7)	13 (4.7)
Gastrointestinal disorders	1 (0.6)	7 (6.2)	8 (2.9)
Abdominal pain	0 (0.0)	2 (2.0)	2 (0.7)
Gastroenteritis	1 (0.6)	1 (1.1)	2 (0.7)
Anorexia	0 (0.0)	1 (1.1)	1 (0.4)
Diarrhea	0 (0.0)	3 (2.9)	3 (1.1)
General disorders	0 (0.0)	1 (1.1)	1 (0.4)
Headache	0 (0.0)	1 (1.1)	1 (0.4)
Respiratory, thoracic, and mediastinal disorders	0 (0.0)	2 (2.0)	2 (0.7)
Cough	0 (0.0)	2 (2.0)	2 (0.7)
Skin and subcutaneous tissue disorders	0 (0.0)	1 (1.1)	1 (0.4)
Skin eruption	1 (0.6)	0 (0.0)	1 (0.4)

### 3.5 Change in biochemistry parameters between day 0 and day 7

**FIGURE 1:**
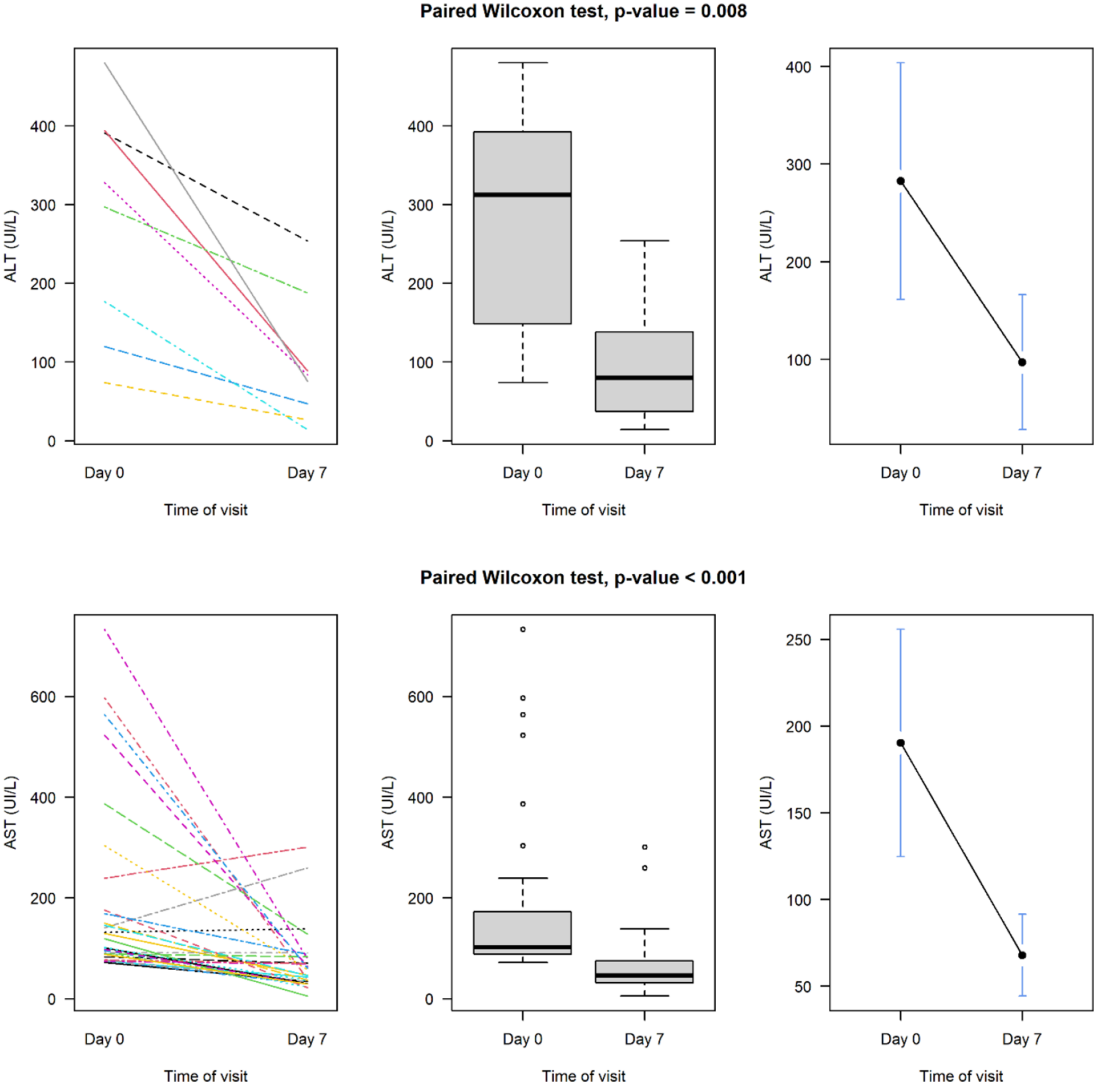
Evolution of liver function tests in patients whose ALT and AST were increased before treatment.

In general, the median biochemical values of biochemistry parameters on day 7 after PA treatment were low compared with the baseline values on day 0 (Figures S1–S3). All the six biochemistry parameters on day 7 post‐treatment were significantly low compared with the baseline values on day 0 among participants. Figure 1 showed what happened to the patients with increased LFTs before treatment. All patients who had elevated ALT prior to study drug experienced a significant decrease in ALT values at day 7. Likewise, patients who had elevated AST prior to study drug showed a significant decrease in AST values on day 7. But individually, there were three patients, who did not have a decrease in AST levels.

## 4 DISCUSSION

This current study was conducted in the mindset to collect safety data to support the scale‐up of PA access in Burkina Faso as well as other developing countries where routine pharmacovigilance is limited. To address this growing need for interest of safety data on PA, this study showed that PA was clinically and biologically well tolerated when used in real‐life conditions in population without clinical liver disorders. Our findings are consistent with previous studies and most frequently observed AEs were similar to these previous studies and malaria symptoms.^13^, ^14^, ^16^, ^19^, ^28^ This study supports what has been observed with PA safety up until now.

In June 2017, PA was added to the WHO Model List of Essential Medicines and Model List of Essential Medicines for Children.^12^ After this adoption, WHO recommended that the deployment of PA should be conducted under a pharmacovigilance system as required for the introduction of all new drugs.^1^ In October 2019, WHO stated that malaria‐endemic countries should consider including PA in their national treatment guidelines, procure it, and monitor its safety and efficacy. Furthermore, in malaria‐endemic countries like Burkina Faso, where SMC with sulfadoxine‐pyrimethamine plus amodiaquine is adopted and implemented since 2016, it is of great interest to assess potential candidates among the registered antimalarial drugs (such as PA) for the diversification of first‐line antimalarial drugs ^9^and because in such area of SMC, ASAQ one of the first‐line therapy should no longer be used.

In this study, the findings did not show the occurrence of serious AEs related to PA treatment during the study period. These findings assert for the wide use of PA in the routine healthcare systems in malaria‐endemic areas. In 2012, PA tablets were granted a positive scientific opinion by the European Medicines Agency (EMA), but with a restricted label, mainly due to concerns over potential hepatic risk of the pyronaridine component, the number of children under 5 years of age treated in the program, and safety, especially with repeated dosing. These restrictions were lifted in 2015 after being reviewed by the EMA of the interim results of the WANECAM repeated doses study. Currently, several scientific research data on hepatic events had provided enough evidence to overcome these concerns about the hepatic risk^13^, ^18^ and thus to allow the EMA and WHO to recommend the use of PA tablets and granules for the treatment and re‐treatment of uncomplicated malaria in patients without signs of hepatic injury (including children weighing 5 kg and over).^12^

The incidence of suspected AEs related to PA reported in our study was relatively low (4.3 per 1000) and most of them were classified as moderate. By investigating the timing of occurrence and type of AEs reported in our study, it is noticeable that most events reported seemed to be related more to malaria than to the intake of PA. In this study, about 5.2% of patients included in the active group reported an AE. This rate was lower than those reported in the previous randomized clinical trial (RCT) carried out among the African or Asian population.^13^, ^14^, ^16^, ^29^, ^30^, ^31^, ^32^, ^33^ However, the type of AEs reported in our study is similar to those reported in CEM study. Indeed, the vomiting rate reported in our study (4.3%) was in agreement (4.2%) with those reported in the CANTAM study assessing in real‐world the safety, tolerability, and effectiveness of PA in malaria patients in five African countries.^19^ Moreover, as revealed in other studies, emesis was more frequent among young patients (less than 15 kg body weight).^19^ This supports the WHO's recent point that there is more vomiting in young children with PA than with other ACTs.

Several reasons could explain this low rate including the design of the study, which consisted of self‐reporting of adverse events. Indeed the latter could not have the same effectivity in detecting adverse events as that achieved in phase 2 and 3 clinical trials. In addition, in this study, it was found that more than 92% of reported AEs were graded as moderate. These findings would suggest that, the AEs with mild severity were not spontaneously reported by the population to the HCWs. The main difference could be due to the fact that safety assessment is more stringent (in particular more visits) in phases II/III studies (more than 50% of the patients' report AEs) than in phase IV studies (5% to 20% of patients reporting AEs). A study assessing the clinical safety of a newly registered antimalarial drug (using the same methodology as described in our study) suggested that the low rate of AE reported in Phase IV study than Phase II and III could be explained by the fact of anxiety of the patient taking a new drug. In addition, the blinded condition in which such drugs are administered may cause an increase of AE reporting.^22^ Moreover, in this study the fact that more than half of the patient was children aged under 5 years of age raised the challenge to obtain reliable safety recall information regarding certain AEs. This could also explain the weakness of the AEs recorded. Another weakness of this study was related to the study design which was a single arm, open‐label, which could lead to report adverse effects for which symptoms presented at the health facility before treatment as AE which were not “new or worsening events” post‐ treatment.

## 5 CONCLUSION

In a context where existing first‐line antimalarial drugs are continuously threatened by the emergence of malaria parasite resistance to antimalarial drugs, the evaluation of the newly registered antimalarial drug, PA, showed a good safety profile in patients of all the age groups in our study. Therefore, PA has deserve of being added to the list of existing first‐line treatments of uncomplicated malaria in Burkina Faso. These findings are useful for the malaria control program in countries for policy decision‐making.

## AUTHORS' CONTRIBUTIONS

TH, TCM, SP, VI, RT, BR, and BF contributed to protocol development. TH, TCM, SP, VI, RT, CA, YWI, TMC, HSF, NAD BR, and BF contributed to data collection and the overall implementation of the study. RT, TH, PS, and YWI worked on data analysis and interpretation of the data. RT and TH drafted the manuscript and all authors read and approved the final manuscript.

## FUNDING INFORMATION

The study was sponsored by the INDEPTH Network as part of the funding for the INESS program.

## DISCLOSURE

The authors report no conflicts of interest in this work.

## ETHICS APPROVAL STATEMENT

The protocol was approved by the institutional ethics committees of the “Institut de Recherche en Sciences de la Santé, Bobo Dioulasso, Burkina Faso (deliberation n° 20‐2017/CEIRES),” and the Ethics Committee for Health Research of Burkina Faso (2017‐6‐075/ CERS).

## PATIENT CONSENT STATEMENT

Written informed consent was obtained from all patients or legal guardians before performing any study‐related activity.

## Supporting information


Appendix S1

## Data Availability

All data generated or analyzed during this study are available at the Clinical Research Unit of Nanoro (CRUN) data repository and shareable upon request addressed to the head of the CRUN.

## References

[1] World Health Organization . Guidelines for the Treatment of Malaria. 3rd ed. 2015. Accessed September 16, 2018. http://apps.who.int/iris/bitstream/10665/162441/1/9789241549127_eng.pdf

[2] World Health Organization . Global Technical Strategy for Malaria 2016–2030. Global Malaria Programme. 2015. Accessed September 4, 2018. http://apps.who.int/iris/bitstream/handle/10665/176712/9789241564991_eng.pdf;jsessionid=66E6DA665C88369AF0BA3A99E8525283?sequence=1

[3] World Health Organization . The Importance of Pharmacovigilance ‐ Safety Monitoring of Medicinal Products. 2002. Accessed April 11, 2018. http://apps.who.int/medicinedocs/pdf/s4893e/s4893e.pdf

[4] Kuemmerle A , Dodoo ANO , Olsson S , Van Erps J , Burri C , Lalvani PS . Assessment of global reporting of adverse drug reactions for anti‐malarials, including artemisinin‐based combination therapy, to the WHO Programme for International Drug Monitoring. Malar J. 2011;10:57. doi:10.1186/1475-2875-10-57 21388536 PMC3063823

[5] Olsson S , Pal SN , Stergachis A , Couper M . Pharmacovigilance activities in 55 low‐ and middle‐income countries. Drug Saf. 2010;33(8):689‐703. doi:10.2165/11536390-000000000-00000 20635827

[6] Olsson S , Pal SN , Dodoo A . Pharmacovigilance in resource‐limited countries. Expert Rev Clin Pharmacol. 2015;8(4):449‐460. doi:10.1586/17512433.2015.1053391 26041035

[7] Elshafie S , Zaghloul I , Roberti AM . Pharmacovigilance in developing countries (part I): importance and challenges. Int J Clin Pharm. 2018;40:758‐763. doi:10.1007/s11096-017-0570-z 29248988

[8] Ministère de la Santé/Programme National de Lutte contre le Paludisme . Directives Nationales Pour La Prise En Charge Du Paludisme Au Burkina Faso. Programme National de Lutte contré le Paludisme (PNLP); 2017.

[9] World Health Organization . WHO Policy Recommendation: Seasonal Malaria Chemoprevention (SMC) for Plasmodium Falciparum Malaria Control in Highly Seasonal Transmission Areas of the Sahel Sub‐Region in Africa. World Health Organization; 2015. Accessed May 23, 2018. http://www.who.int/malaria/publications/atoz/who_smc_policy_recommendation/en/

[10] Gansané A , Moriarty LF , Ménard D , et al. Anti‐malarial efficacy and resistance monitoring of artemether‐lumefantrine and dihydroartemisinin‐piperaquine shows inadequate efficacy in children in Burkina Faso, 2017–2018. Malar J. 2021;20(1):48. doi:10.1186/s12936-021-03585-6 33468147 PMC7816451

[11] Rasmussen C , Ringwald P . Is there evidence of anti‐malarial multidrug resistance in Burkina Faso? Malar J. 2021;20(1):320. doi:10.1186/S12936-021-03845-5 34281562 PMC8287766

[12] World Health Organization . The use of artesunate‐pyronaridine for the treatment of uncomplicated malaria. *WHO/HTM/GMP/2019.13* . 2019. https://www.who.int/publications‐detail/use‐of‐artesunate‐pyronaridine‐for‐the‐treatment‐of‐uncomplicated‐malaria

[13] Pryce J , Hine P . Pyronaridine‐artesunate for treating uncomplicated *Plasmodium falciparum* malaria. Cochrane Database Syst Rev. 2019;1:CD006404. doi:10.1002/14651858.CD006404.pub3 30620055 PMC6353203

[14] Sagara I , Beavogui AH , Zongo I , et al. Pyronaridine–artesunate or dihydroartemisinin–piperaquine versus current first‐line therapies for repeated treatment of uncomplicated malaria: a randomised, multicentre, open‐label, longitudinal, controlled, phase 3b/4 trial. Lancet. 2018;391(10128):1378‐1390. doi:10.1016/S0140-6736(18)30291-5 29606364 PMC5889791

[15] Ramharter M , Kurth F , Schreier AC , et al. Fixed‐dose pyronaridine‐artesunate combination for treatment of uncomplicated falciparum malaria in pediatric patients in Gabon. J Infect Dis. 2008;198(6):911‐919. doi:10.1086/591096 18694333

[16] Duparc S , Borghini‐Fuhrer I , Craft CJ , et al. Safety and efficacy of pyronaridine‐artesunate in uncomplicated acute malaria: an integrated analysis of individual patient data from six randomized clinical trials. Malar J. 2013;12(1):70. doi:10.1186/1475-2875-12-70 23433102 PMC3598551

[17] Sagara I , Beavogui AH , Zongo I , et al. Safety and efficacy of re‐treatments with pyronaridine‐artesunate in African patients with malaria: a substudy of the WANECAM randomised trial. Lancet Infect Dis. 2016;16(2):189‐198. doi:10.1016/S1473-3099(15)00318-7 26601738 PMC4726763

[18] Compaoré YD , Zongo I , Somé AF , et al. Hepatic safety of repeated treatment with pyronaridine‐artesunate versus artemether–lumefantrine in patients with uncomplicated malaria: a secondary analysis of the WANECAM 1 data from Bobo‐Dioulasso, Burkina Faso. Malar J. 2021;20(1):64. doi:10.1186/s12936-021-03593-6 33514368 PMC7847156

[19] Lutete GT , Mombo‐Ngoma G , Assi S‐B , et al. Pyronaridine–artesunate real‐world safety, tolerability, and effectiveness in malaria patients in 5 African countries: a single‐arm, open‐label, cohort event monitoring study. PLoS Med. 2021;18(6):e1003669. doi:10.1371/JOURNAL.PMED.1003669 34129601 PMC8205155

[20] Derra K , Rouamba E , Kazienga A , et al. Profile: nanoro health and demographic surveillance system. Int J Epidemiol. 2012;41(5):1293‐1301. doi:10.1093/ije/dys159 23045201

[21] Rouamba T , Sondo P , Derra K , et al. Optimal approach and strategies to strengthen pharmacovigilance in sub‐saharan africa: a cohort study of patients treated with first‐line artemisinin‐based combination therapies in the nanoro health and demographic surveillance system, Burkina Faso. Drug Des Devel Ther. 2020;14:1507‐1521. doi:10.2147/DDDT.S224857 PMC7174163 32368010

[22] Baiden R , Oduro A , Halidou T , et al. Prospective observational study to evaluate the clinical safety of the fixed‐dose artemisinin‐based combination Eurartesim® (dihydroartemisinin/piperaquine), in public health facilities in Burkina Faso, Mozambique, Ghana, and Tanzania. Malar J. 2015;14(160):1‐7. doi:10.1186/s12936-015-0664-9 25885858 PMC4405867

[23] Rouamba T , Nakanabo‐Diallo S , Derra K , et al. Socioeconomic and environmental factors associated with malaria hotspots in the Nanoro demographic surveillance area, Burkina Faso. BMC Public Health. 2019;19(249):1‐14. doi:10.1186/s12889-019-6565-z 30819132 PMC6396465

[24] Ministère de la Santé/Direction Générale des Etudes et des Statistiques Sectorielles . Annuaire Statistique 2018. Ministère de la Santé; 2019.

[25] Rouamba T , Sondo P , Yerbanga IW , et al. High adherence level to artemisinin‐based combination therapies in rural settlement 11 years after their introduction in the health system, Nanoro, Burkina Faso. Patient Prefer Adherence. 2019;13:371‐380. doi:10.2147/PPA.S190927 30880921 PMC6402368

[26] International Conference on Harmonisation of Technical Requirements for Registration of Pharmaceuticals for Human Use . ICH Harmonised Tripartite . Guideline Post‐Approval Safety Data Management: Definitions and Standards for Expedited Reporting (ICH‐E2D); 2003. Accessed July 15, 2018. https://www.ich.org/fileadmin/Public_Web_Site/ICH_Products/Guidelines/Efficacy/E2D/Step4/E2D_Guideline.pdf

[27] Safety Reporting and Pharmacovigilance|NIH: National Institute of Allergy and Infectious Diseases. Accessed October 12, 2021. https://www.niaid.nih.gov/research/dmid‐safety‐reporting‐pharmacovigilance

[28] Rueangweerayut R , Phyo AP , Uthaisin C , et al. Pyronaridine‐artesunate versus mefloquine plus artesunate for malaria. N Engl J Med. 2012;366(14):1298‐1309. doi:10.1056/NEJMoa1007125 22475593

[29] Kayentao K , Doumbo OK , Pénali LK , et al. Pyronaridine‐artesunate granules versus artemether‐lumefantrine crushed tablets in children with *Plasmodium falciparum* malaria: a randomized controlled trial. Malar J. 2012;11(1):364. doi:10.1186/1475-2875-11-364 23113947 PMC3566922

[30] Leang R , Canavati SE , Khim N , et al. Efficacy and safety of pyronaridine‐artesunate for treatment of uncomplicated *Plasmodium falciparum* malaria in western Cambodia. Antimicrob Agents Chemother. 2016;60(7):3884‐3890. doi:10.1128/AAC.00039-16 26926629 PMC4914696

[31] Leang R , Khim N , Chea H , et al. Efficacy and safety of pyronaridine‐artesunate plus single‐dose primaquine for the treatment of malaria in western Cambodia. Antimicrob Agents Chemother. 2019;63(10). doi:10.1128/AAC.01273-19 PMC6761550 31358594

[32] Roth JM , Sawa P , Makio N , et al. Pyronaridine‐artesunate and artemether‐lumefantrine for the treatment of uncomplicated *Plasmodium falciparum* malaria in Kenyan children: a randomized controlled non‐inferiority trial. Malar J. 2018;17(1):199. doi:10.1186/s12936-018-2340-3 29764419 PMC5952621

[33] Tshefu AK , Gaye O , Kayentao K , et al. Efficacy and safety of a fixed‐dose oral combination of pyronaridine‐artesunate compared with artemether‐lumefantrine in children and adults with uncomplicated *Plasmodium falciparum* malaria: a randomised non‐inferiority trial. Lancet (London, England). 2010;375(9724):1457‐1467. doi:10.1016/S0140-6736(10)60322-4 20417857

