# Supplementary material for: Prospective observational study to evaluate the clinical and biological safety profile of pyronaridine–artesunate in a rural health district in Burkina Faso
Source: Pharmacol Res Perspect. 2022 Jul 19;10(4):e00987. doi: 10.1002/prp2.987 (PMC9297024; doi:10.1002/prp2.987)
Supplement: Supplementary file 1 — Appendix S1 [file PRP2-10-e00987-s001.docx]

# **Supplementary materials**

Supplementary material 1: Description of Serious adverse events occurring during the study period

| Case 1 | Female patient, 02 years old, with no particular medical or surgical history, included in the study on August 09, 2018 with a diagnosis of severe malaria with severe anemia. During hospitalization, the patient presented a good clinical course after blood transfusion and parental antimalarial treatment. On August 15, 2018, we noted the disappearance of fever. Microscopy performed the same day was negative and the hemoglobin level was 7.4 g/dl. So, she was discharged on August 15, 2018 under oral treatment. The clinical examination performed on September 06, 2018 on the 28th day found a good health status with a hemoglobin level of 10.5 g/dl. Faced with these results we declared the patient fully recovered. |
| --- | --- |
| Case 2 | Male patient, 03 years old, with no particular medical or surgical history, included in the study on August 23, 2018 and diagnosed with severe anemia (Hb: 3.9 g/dl). The patient was hospitalized the same day and a blood transfusion was performed. The patient's clinical condition after the blood transfusion was stable. During the hospitalization, the patient showed a good clinical course and the hemoglobin level realized on August 31, 2018 was 7.6 g/dl. Therefore, she was discharged from the health center the same day with oral treatment. A clinical follow-up examination performed on October 16, 2018, noted good clinical health and normalization of the hemoglobin level to 10.5 g/dl. We therefore declared this SAE closed. |
| Case 3 | Male patient, 03 years old, with no particular medical or surgical history, included in the study on August 23, 2018 who presented on September 12, 2018 with severe malaria with severe anemia (4.3 g/dl). A blood transfusion was performed the same day. The patient's clinical condition after the blood transfusion was stable. A control of biochemical values performed on September 20, 2018, showed elevated transaminase values with AST (SGOT) at 149 UI/L and ALT (SGPT) at 224 UI/L but with normal total bilirubin. The clinical examination noted a good general condition without jaundice, asthenia or hepatomegaly. The rest of the clinical examination was also good. With these findings, another biochemical assessment was performed on September 24, 2018, which showed a decreased transaminase level with AST (SGOT) at 45 UI/L and ALT (SGPT) at 102 UI/L. Hepatitis A, B, and C tests performed on September 24, 2018 were negative. A pediatric consultation was also done on the same day that evoked a diagnosis of malaria-induced liver cytolysis. According to the pediatrician, these types of liver cytolysis resolve spontaneously within a few weeks. We therefore planned to monitor the evolution of transaminases until these values were normalized. Monitoring of transaminase values on October 15, 2018, found normalization of these values with AST (SGOT) at 37 UI/L and ALT (SGPT) at 32 UI/L and total bilirubin at 6.95 μmol/L. We therefore declared the patient fully recovered. |
| Case 4 | Female patient, 10 years old, with no particular medical or surgical history, except a sickle cell trait (hemoglobin electrophoresis: AS), included in the study on February 07, 2018 and diagnosed with splenic abscess on February 19, 2018. The patient was hospitalized the same day. Chest X-ray performed on February 20, 2018 showed left pleurisy and abdominal ultrasound performed on the same day showed homogeneous hepatomegaly, multiple splenic abscesses with ascites of low abundance. Blood cultures performed on February 19, 2018 and March 01, 2018 respectively were negative. Follow-up of the patient's condition noted a good evolution and the abdominal ultrasound performed on March 10, 2018 showed heterogeneous splenomegaly with persistence of 2 abscesses at the apical pole, regression of hepatomegaly and persistence of ascites of low abundance. Another abdominal ultrasound performed on May 05, 2018, showed a restitution of the size of the spleen, which was homogeneous. This examination also noted the absence of hepatomegaly and ascites. We therefore declared the patient fully recovered. |
| Case 5 | Male patient, 03 years old, with no particular medical or surgical history, included in the study on February 22, 2018, with a diagnosis of a second-degree superficial burn of approximately 45% of the body surface (arms, back, buttocks, abdomen, thighs, and genitals). The patient was admitted to the hospital's surgical ward and emergency care including dressing, rehydration, analgesics and antibiotics was initiated. After these treatments, the patient's condition was stable. However, two days after hospitalization (February 29, 2018), the patient presented with an episode of fever (axillary temperature at 39.2ºC) but cardiac and pulmonary examinations were normal. The clinical course worsened with a progressive alteration of his general condition and an occurrence of generalized convulsions without fever (T° 37º0 C) on March 05, 2018. A diagnosis of septic shock was established and the patient was transferred to the resuscitation department of the pediatric ward of the hospital for optimal management. Clinical examination found coma (stage 4) associated with decerebration rigidity and respiratory distress that motivated oxygenation (at 2L/min).  The clinical course under oxygen was complicated by the occurrence of cardiorespiratory failure. Resuscitative measures (cardiac massage and manual ventilation) were performed without success. Cardiopulmonary arrest with reflexes mydriasis occurred and death was declared on March 05, 2018. |
| Case 6 | Female patient, 63 years old, with no particular medical or surgical history, included in the study on August 24, 2018. The first dose of PYRAMAX was administered by the study nurse. The second and third doses were also well administered at home according to the mother. The clinical examination from day 7 to September 07, 2018 was good. On September 22, 2018, the field worker visited the patient's home for the end of follow-up visit (day 28). It was during this visit that she was informed that the patient had died. From the investigations performed, we understood that the patient was hospitalized at Hospital from September 13 to 20, 2018. The summary of the patient's medical history from hospital to death according to the Hospital medical record is as follows:  The patient reportedly had tonsillitis on September 09, 2018 untreated as it was considered not severe. She consulted on September 13, 2018 for vomiting, dizziness, throat pain. The clinical and paraclinical examination on the same day found tonsillitis complicated by acute glomerulonephritis, acute renal failure (urea: 314 mg / dl creatinine: 10.3 mg/dL), bronchopneumonia and anemia at 5.8 g/dL. With this diagnosis, the patient was hospitalized with parental treatment. During the hospitalization, it was noted a worsening of the patient's clinical condition with the occurrence of heart failure and respiratory distress that led to the death of the patient on September 20, 2018 at about 19:50. |

**Supplementary material** 2:

**
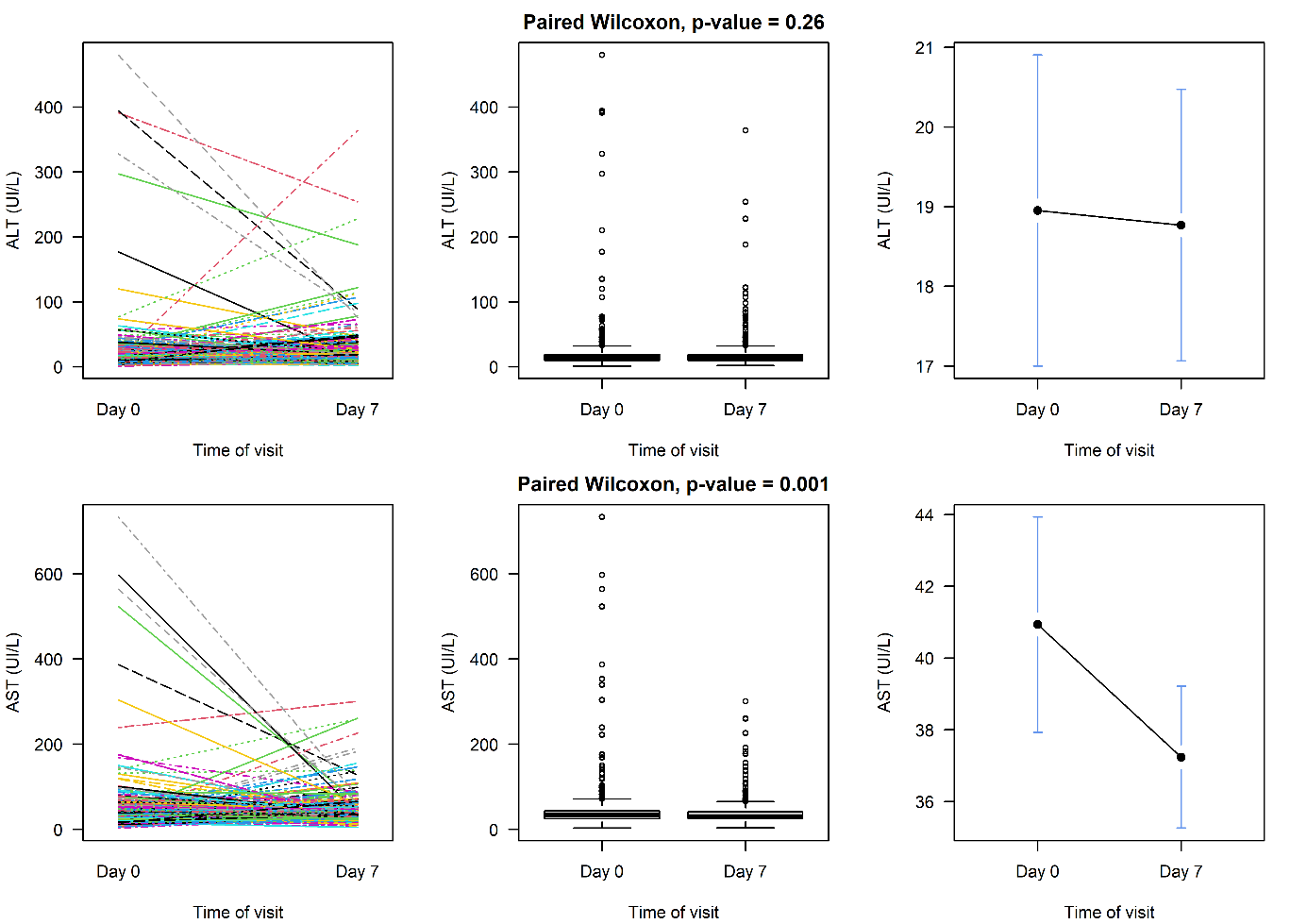
**

**Figure S1**. Change in aminotransferase (ALT) and aspartate aminotransferase (AST) values between day-0 and day-7


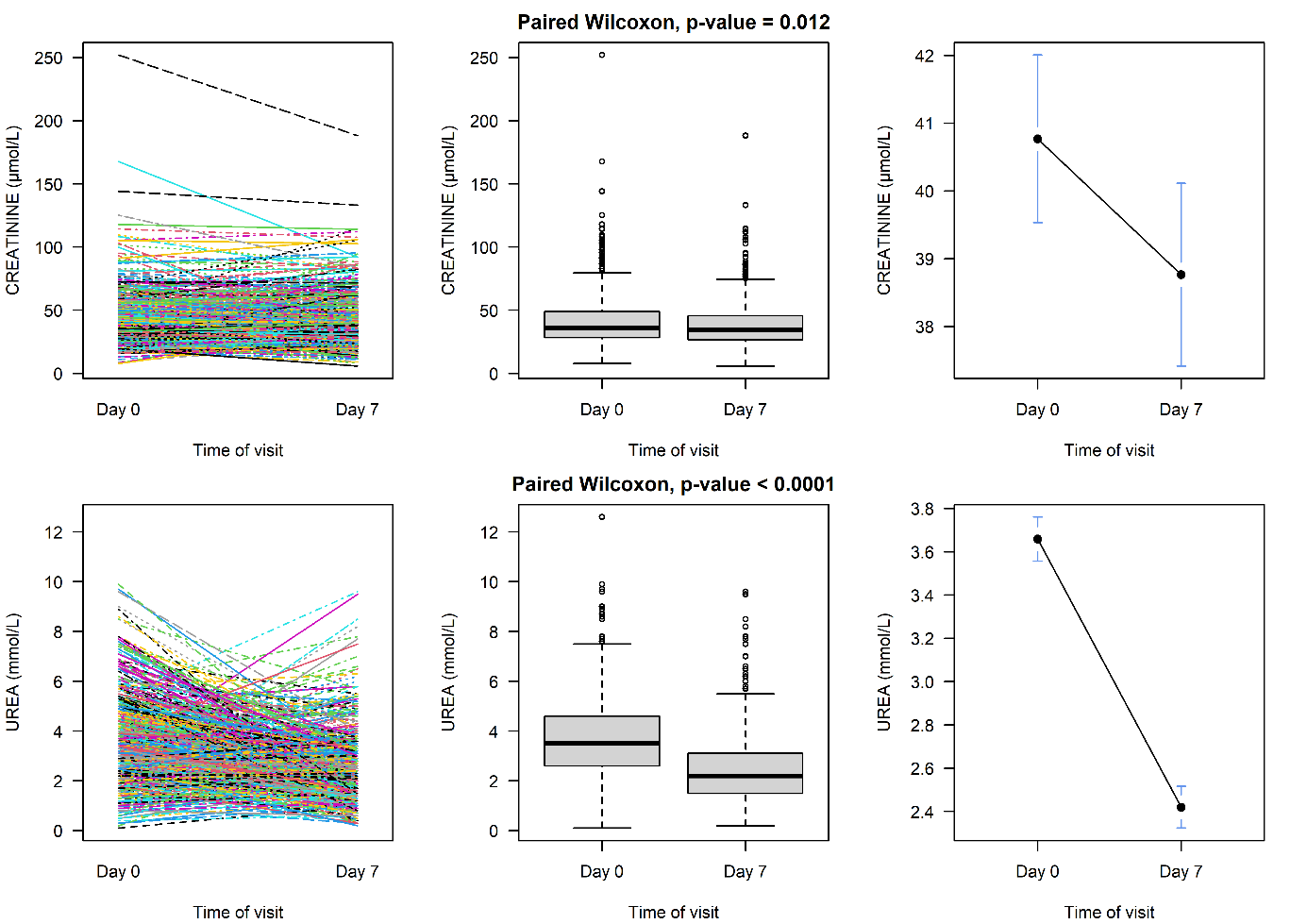


**Figure S2**. Change in creatinine and urea values between day-0 and day-7

**
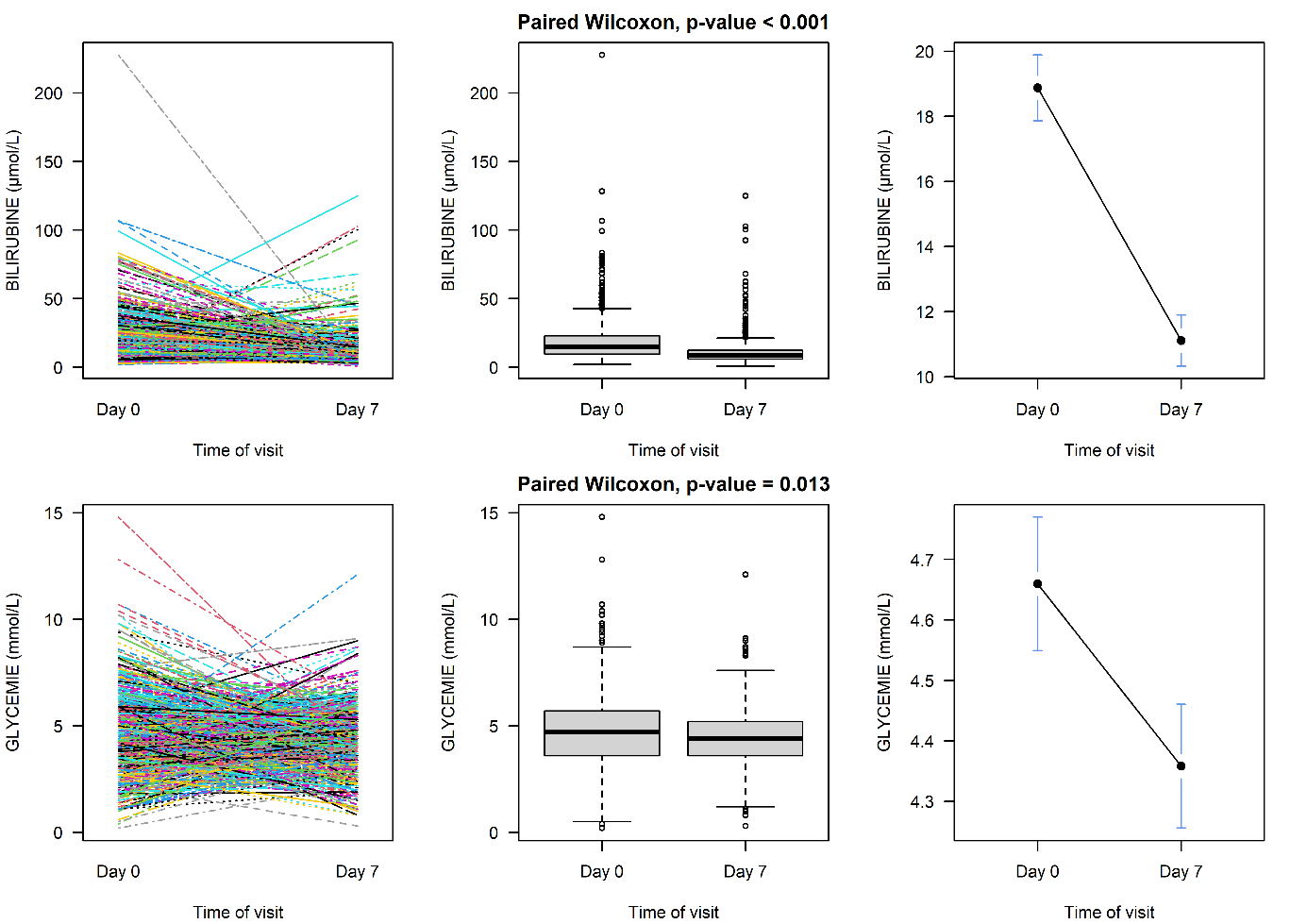
**

**Figure S3**. Change in glycaemia and bilirubin values between day-0 and day-7
